# Exomphalos Major Associated with Thoracic Teratoma and Hamartoma of Left Leg: A Rare Association

**Published:** 2014-07-10

**Authors:** KN Rattan, Poonam Dalal, Mohit Gupta

**Affiliations:** 1 Department of Pediatric Surgery, Pt. B. D. Sharma, PGIMS, Rohtak; 2 Department of Pediatrics, Pt. B. D. Sharma, PGIMS, Rohtak

**Dear Sir**

A 2-day-old, full term (38 weeks), appropriate for gestational age, female baby was delivered vaginally to 22-year-old primigravida at peripheral health facility. The baby did not cry at birth, required positive pressure ventilation for 3 minutes at birth and Apgar score at 1 and 5 minutes after birth were 3/10 and 6/10 respectively. No features of hypoxic ischemic encephalopathy were noticed. The newborn was referred for further investigation and treatment to tertiary health center. There was no history of any drug intake or radiation exposure in mother during antenatal period. On examination, a mass of 4.3 cm × 4 cm × 2.5 cm with two skin appendages was found to be attached to the lower part of the chest wall of the baby (Fig. 1). An exomphalos major and hamartoma of size 3.2 X 3.5 cm in left lower leg was present in the neonate. The liver was protruding into the exomphalos sac that was confirmed on abdominal sonography. Ultrasonography of chest, skull, spine and abdomen, apart from exomphalos were normal. Echocardiography revealed small ventricular septal defect (VSD). 

The thoracic mass was excised by elliptical incision after ligating the feeding vessels without any complications. The primary repair of exomphalos major was also done simultaneously. The contents of sac were part of liver and small bowel. Histopathological examination labeled the separated mass as teratoma. The postoperative period was uneventful and baby was discharged after 7 days. The baby is growing well in the follow up. The small VSD closed spontaneously and hamartoma did not increase in size during the follow up period. Associated malformations are not rare with omphalocele, but teratoma associated with congenital omphalocele is extremely rare. Only 11 cases of teratoma associated with omphalocele are reported in the literature hitherto. The teratoma in the reported cases was present as a part of omphalocele sac in six cases and five cases had umbilical cord teratoma [1-5]. Again, hamartoma in association with omphalocele major other than mesenchymal hamartoma of liver is extremely rare [6,7]. So, we are reporting a rare case of exomphalos major associated with thoracic teratoma and hamartoma of left lower leg.

**Figure:**
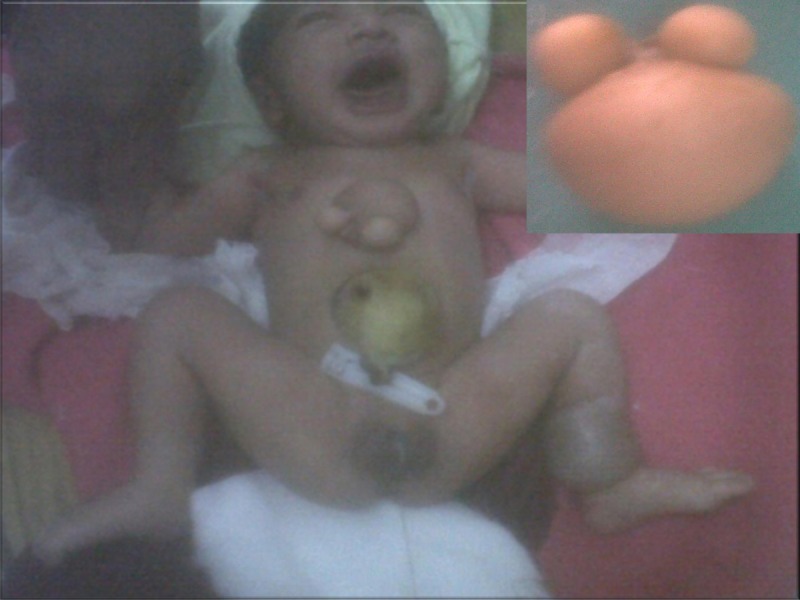
Figure 1: Neonate with omphalocele major, teratoma and hamartoma of left leg and inset showing separated mass, later on diagnosed as teratoma.

## Footnotes

**Source of Support:** Nil

**Conflict of Interest:** None

